# Impact of lesion preparation-induced calcified plaque defects in vascular intervention for atherosclerotic disease: in silico assessment

**DOI:** 10.1007/s10237-024-01923-6

**Published:** 2025-01-21

**Authors:** Jonas Sogbadji, Karim Kadry, Gianluca Poletti, Francesca Berti, Elazer R. Edelman, Farhad R. Nezami

**Affiliations:** 1 Institute for Medical Engineering and Science, Massachusetts Institute of Technology, 77 Massachusetts Ave, Cambridge, MA 02139 USA; 2 LaBS-Department of Chemistry, Materials and Chemical Engineering, Politecnico di Milano, Piazza Leonardo da Vinci 32, Milano, 20133 Italy; 3 Cardiovascular Division, Brigham and Women’s Hospital, Harvard Medical School, Boston, MA 02115 USA; 4 Cardiac Surgery Division, Brigham and Women’s Hospital, Harvard Medical School, Boston, MA 02115 USA

**Keywords:** Finite element analysis, Computational biomechanics, Virtual angioplasty, Vessel preparation, Intravascular lithotripsy, Stent expansion

## Abstract

Percutaneous coronary interventions in highly calcified atherosclerotic lesions are challenging due to the high mechanical stiffness that significantly restricts stent expansion. Intravascular lithotripsy (IVL) is a novel vessel preparation technique with the potential to improve interventional outcomes by inducing microscopic and macroscopic cracks to enhance stent expansion. However, the exact mechanism of action for IVL is poorly understood, and it remains unclear whether the improvement in-stent expansion is caused by either the macro-cracks allowing the vessel to open or the micro-cracks altering the bulk material properties. In silico models offer a robust means to examine (a) diverse lesion morphologies, (b) a range of lesion modifications to address these deficiencies, and (c) the correlation between calcium morphology alteration and improved stenting outcomes. These models also help identify which lesions would benefit the most from IVL. In this study, we develop an in silico model of stent expansion to study the effect of macro-crack morphology on interventional outcomes in clinically inspired geometries. Larger IVL-induced defects promote more post-stent lumen gain. IVL seems to induce better stenting outcomes for large calcified lesions. IVL defects that split calcified plaque in two parts are the most beneficial for stenting angioplasty, regardless of the calcified plaque size. Location of the IVL defect does not seem to matter with respect to lumen gain. These findings underscore the potential of IVL to enhance lesion compliance and improve clinical outcomes in PCI. The macroscopic defects induced by IVL seem to have a substantial impact on post-stent outcomes.

## Introduction

Atherosclerosis is the most prevalent form of obstructive vascular disease and the leading cause of mortality worldwide (Pahwa and Jialal 2023). Percutaneous coronary intervention (PCI) has become the gold standard for minimally invasive treatments (Hill et al. 2020a) with tremendous impact. Yet, PCI proves less effective in those cases involving coronary artery calcification (CAC), which stiffen the whole artery (Kereiakes et al. 2021), thereby preventing optimal stent expansion and reducing the intervention efficacy (Dong et al. 2018, Tsutsumi et al. (2008)). Indeed, CAC is responsible for stent malapposition, which has been already addressed as a major cause for late stent thrombosis (Hill et al. 2020a,Hemetsberger et al. (2021)), stent underexpansion (strong predictor for adverse events such as in-stent restenosis and thrombosis (Buccheri et al. 2016, Hill et al. (2020a)) or non-optimal drug delivery for drug-eluting stents (Edelman et al. 2022; Buccheri et al. 2016; Hill et al. 2020a; Hess et al. 2021; Brinton et al. 2019; Kereiakes et al. 2021; Kaul et al. 2020). Hemetsberger et al. (2021) studied patients undergoing PCI and found that those with moderate to severe coronary calcification had significantly higher rates of target lesion failure (13.5% vs 8.4%) and stent thrombosis (2.1% vs 0.2%) compared to those with mild to no calcification. These findings underscore the importance of understanding and addressing calcified lesion morphology to improve stenting outcomes.

Inspired by current clinical routine in the treatment of renal calculi, intravascular lithotripsy (IVL) (Shockwave Medical, Santa Clara, CA) is an innovative approach that utilizes a balloon angioplasty catheter shaft equipped with lithotripsy emitters, which generate localized acoustic pressure waves that travel radially through the arterial wall, undermining the structure of calcified plaques (Hess et al. 2021, Brinton et al. (2019), Kereiakes et al. (2021)) without affecting healthy tissue and surrounding vessels (Kereiakes et al. 2021). In that context, a spectrum of morphological and mechanical alterations that impact vessel compliance is typically observed:

- Macroscopic defects that create calcified plaque mobility within the lesion and are seen in optical coherence tomography (OCT) images (Kereiakes et al. 2021),
- Microscopic defects that collectively reduce the stiffness of the lesion, typically seen in micro-CT or histology (Kereiakes et al. 2021)

This vessel preparation technique causes the mechanical properties of calcified plaques to degrade (lesion is therefore softer), which enhances the effectiveness of balloon or stent placement and deployment (Kereiakes et al. 2021). For this reason, IVL has emerged as an effective and safe technique for treating calcified lesions in coronary and peripheral artery disease and optimize endovascular interventions. Multiple studies have demonstrated high procedural success rates, with significant reductions in diameter stenosis and minimal complications (Oliveira et al. 2024; Wong et al. 2022; Mhanna et al. 2021; Choksi et al. 2023). In coronary applications, IVL achieved clinical success in 95.4% of cases and angiographic success in 97% (Mhanna et al. 2021). For peripheral artery disease, IVL resulted in a diameter stenosis reduction of 59.3% with rare vascular complications (Wong et al. 2022). Across studies, IVL demonstrated effectiveness in severely calcified lesions, with success rates often reaching 100% (Choksi et al. 2023; Oliveira et al. 2024). Studies have shown high procedural success rates and low incidence of major adverse cardiovascular events (MACE) at 30 days post-IVL (Kereiakes et al. 2020; Saito et al. 2022; Hill et al. 2020b). One-year follow-up data demonstrate sustained safety and efficacy, with low rates of MACE, target lesion revascularization, and stent thrombosis (Saito et al. 2022; Kereiakes et al. 2022). IVL has also shown promise in treating stent failure, with a procedural success rate of 78.4% and a one-year MACE rate of 15.7% (Kuzemczak et al. 2023). However, complications can occur, including device malfunction, balloon dislodgment, and rare cases of coronary dissection (Chugh et al. 2021). Factors such as ostial disease and lesion length may influence long-term outcomes (Kuzemczak et al. 2023). Overall, IVL appears to be a safe and effective option for preparing calcified coronary lesions, but continued vigilance and monitoring are warranted.

Despite the clinical enthusiasm and proven reliability of IVL (Kereiakes et al. 2021), the precise mechanisms by which these multiscale structural calcium modifications contribute to improved post-stent clinical outcomes remain poorly understood.

To address this gap, given the substantial range and extent of lesions, it is a formidable challenge to manage their modification in clinical and preclinical models. However, in silico lesion models, combined with virtual angioplasty, provide a valuable tool for investigating different lesion morphologies and modifications, helping us understand the role of IVL-induced modifications on the improvement of stenting outcomes (Athanasiou et al. 2019). While (McAteer and Evan 2008) have outlined the benefits of shock wave lithotripsy (SWL) in effectively removing kidney stones, the impact of IVL-induced morphological changes on calcified plaque and subsequent vessel expansion remains unexplored in existing literature regarding endovascular treatments.

This study aims to investigate the validity of the hypothesis that calcium macro-fractures, induced by IVL and referred herein as IVL-induced defects (IVL-ID), improve vessel expansion during stenting. Initially, we develop a virtual angioplasty platform comprising image-informed parametric models of calcified vessels post-IVL, along with a coronary stent delivery system.

## Materials and methods

We begin by explaining our preliminary morphology analysis, which yielded two geometrical metrics that informed our models of IVL-induced defects. In Sec. 2.1, we introduce our parametric model of crack geometry. Our virtual angioplasty platform is discussed in Sec. 2.2, and our computational configuration is detailed in Sec. 2.3. It is worth noting that as we are interested in the impact of the IVL-IDs on stenting, we are not modeling shockwave propagation and interaction with materials: the artery models we use are post-IVL. We then perform virtual deployment simulations of our generic stent (and inspired by commercial ones) in different parametric geometries inspired by different physiological crack configurations and analyze vessel expansion.

### IVL-induced calcified lesion morphologies

To incorporate clinical realism into our models, we 3D reconstructed and analyzed pre- and post-IVL optical coherence tomography (OCT) pullbacks (Fig. 2a, b). Our post-IVL OCT images are derived from the Disrupt CAD-III study (Hill et al. 2020b). In brief, patients presenting with stable, unstable, or silent ischemia and severely calcified de novo coronary artery lesions undergoing PCI were eligible for enrollment. Target lesions were smaller than 40 mm in length with reference vessel diameters of 2.5 to 4.0 mm. Patients with acute MI and specific complex lesion features were excluded. Complete inclusion and exclusion criteria for the study are listed in Hill et al. (2020b). For each patient, the IVL catheter was delivered over the physician’s choice of a 0.014-inch guidewire. If the catheter could not cross the lesion, adjunctive methods were used. Atherectomy devices and cutting/scoring balloons were not permitted by protocol. The IVL balloon was inflated to 4 atm, delivering 10 pulses followed by balloon inflation to 6 atm. This process was repeated until complete lesion preparation was achieved. Noncompliant balloon dilation was performed before stenting in lesions with residual stenosis larger than 50%. Post-dilatation (greater than 16 atm) was required after stent implantation. OCT was used at three time points: pre-IVL, post-IVL, and post-stent deployment. This was done to assess calcification and evaluate the role of IVL in facilitating stent expansion. In these OCT images, we examined the geometrical characteristics of the calcified lesions and the macroscopic IVL-induced defects. Table 1 shows a general description of the patient and lesion characteristics.

**Table 1:** OCT study details

Statistic	Value
(a) Patient characteristics	
Number of men	202
Number of women	60
Age (men)	$71 \pm 9$
Age (women)	$76 \pm 9$
(b) Lesion characteristics	
Lesion length (mm)	$26 \pm 11$
Calcium length (mm)	$42 \pm 21$
% stenosis diameter (%)	$62 \pm 12$
Calcium arc angle (deg)	$270 \pm 81$
Calcium thickness (mm)	$1 \pm 0.23$

From this analysis, we identified two different types of calcified plaques based on their geometrical extension: distributed ones (when the plaque covers more than half of the circumference of each interested OCT slice) and more localized ones. In this study, the lesion was considered to be composed of both stiff tissue (mostly calcified plaque) and soft tissue (mostly lipidic plaque). The material properties of the lesion are presented in Table 2 and are extracted from Poletti et al. (2022).

**Table 2:** Lesion material properties from the study by Poletti et al. (2022)

Tissue	Material model	Parameters
Lipidic plaque	Neo-Hookean	$C_{10} = 13.3$ KPa
	Hyperelastic plastic	$\sigma _{yield} = 70$ KPa
		incompressible
Calcified plaque	Linear elastic–plastic	$E = 44$ MPa
		$\sigma _{yield} = 480$ KPa
		$$\nu = 0.3$$

**Fig. 1:**
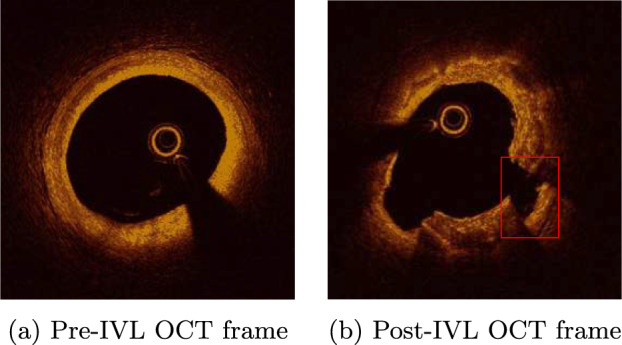
Pre-IVL and post-IVL OCT frame. A cross section of the idealized artery showing the defect highlighted in Fig. 1b can be found in Fig. 3

Drawing inspiration from our OCT analyses, we initially proposed two baseline lesion models, referred to as intact, which are devoid of defects. One model, being representative of the distributed calcification, features a $270^{\circ }$ ($\theta _c=270^{\circ }$) circumferential calcified plaque coverage (consistent with the calcium arc angle value given in Table 1b); while, the other includes a $90^{\circ }$ calcified plaque coverage, as in localized lesions ($\theta _c=90^{\circ }$). Both models maintain a consistent length of $L_c=10mm$, different from the OCT analysis results given in Table 1b for the sake of diminishing the number of meshing elements for the finite element analyses, and thus reduce computational time. The thickness is also the same across all artery models and has the value of $t=1.1mm$ corresponding to the maximum calcified plaque thickness observed in the OCT geometrical analysis (Table 1b).

**Fig. 2:**
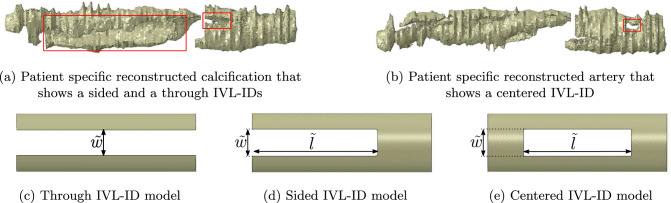
Three IVL-ID morphology models 2c, d and e are based on what is seen after analyses of 3D reconstructed patient-specific coronary arteries from OCT pullbacks: the sided (Fig 2d, right box of Fig 2a), the centered (Fig 2e, box of Fig 2b) and the through (Fig 2c, left box of Fig 2a) IVL-ID models Fig. 2b and a are calcification reconstruction from the same artery

From these intact models, we constructed different, simplified yet representative lesion models with IVL-IDs and modified plaque properties. The defect was created by removing a rectangular volume from the calcified plaque component of the intact models. Here, we defined $\tilde{w}$ and $\tilde{l}$ as the width and the length of the induced defect, respectively (Fig. 2). To facilitate the analysis, we then introduced the non-dimensionalized parameter *w* as follows:

1 $$\begin{aligned} w = \frac{\theta (\tilde{w})}{\theta _c} \end{aligned}$$

**Fig. 3:**
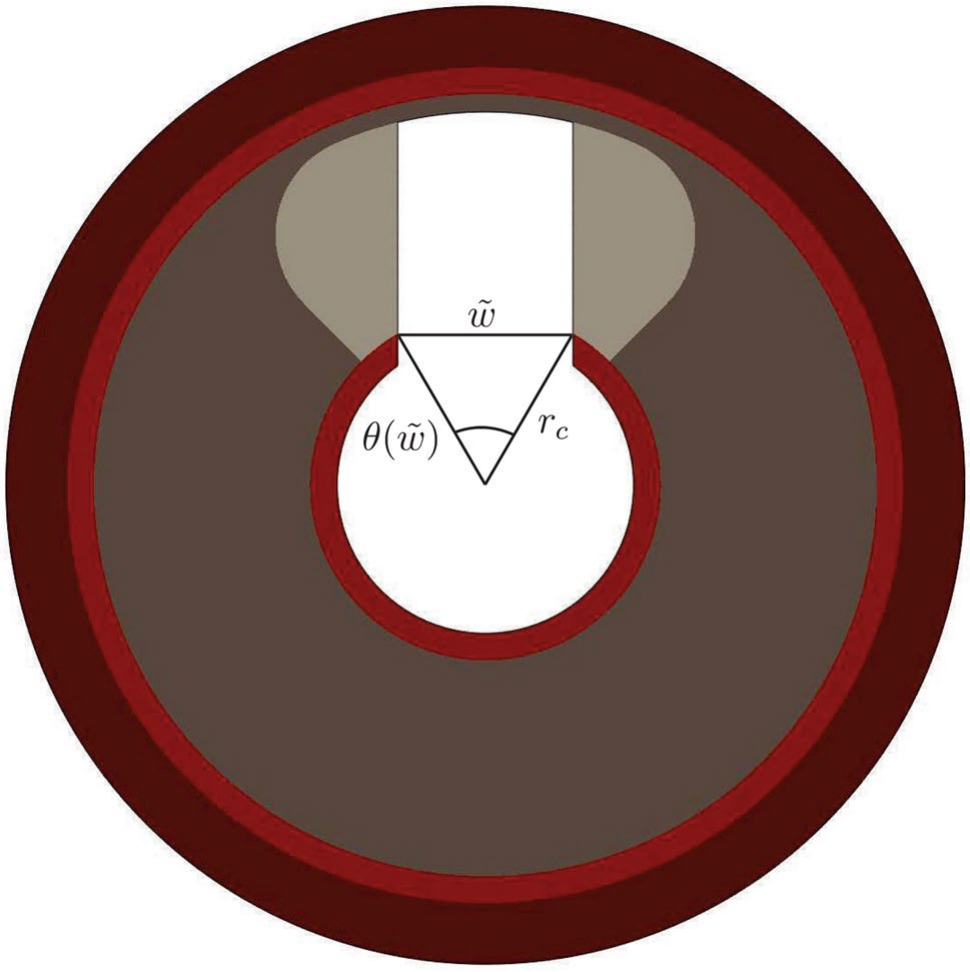
Cross sectional view of one idealized artery model which shows the definition of the non-dimensionalized parameter *w*. Dark red is adventitia, light red is media/intima, dark gray is soft plaque tissue and light gray is hard plaque tissue

With $\theta (\tilde{w})=\arccos \Bigl (1-\frac{\tilde{w}^2}{2r_c^2}\Bigr )$ and $r_c$ being the radial position of the calcium (Fig. 3). $\tilde{w}$ is normalized by the angular opening that the induced defect creates at $r_c$ relative to the angular dimension of the calcium component. To ensure realistic width values based on OCT analyses, *w* examined two values: $\frac{1}{3}$ (modeling the narrow defects) and $\frac{2}{3}$ (modeling the wide defects).

Similarly, we defined the non-dimensionalized parameter *l*:

2 $$\begin{aligned} l=\frac{\tilde{l}}{L_c} \end{aligned}$$

To align with realistic length values observed in OCT analyses, *l* was set as $\frac{1}{3}$ (modeling the short defects) or $\frac{2}{3}$ (modeling the long defects). We chose this binary categorization of the IVL-IDs as we noticed through the OCT study that they do not seem to appear with a preferential morphology (Table 3).

**Table 3:** Possible combinations of *l* and *w*

$N^{\circ }$	*l*	*w*
1	1/3	1/3
2	1/3	2/3
3	2/3	1/3
4	2/3	2/3

We then proposed three distinct lesion sub-models, each tailored to capture a specific IVL-ID morphology observed on OCT pullbacks (Fig. 2).

### Virtual angioplasty platform

The platform comprises the stent, the angioplasty balloon, and several mock-up arteries.

The stent measures 20*mm* in length, with a nominal diameter of 3*mm* and a strut radial thickness of $70\mu m$. Made of Platinum–chromium, the stent exhibits an elasto-plastic mechanical behavior, characterized by plastic behavior described by the following (using the common notations):

3 $$\begin{aligned} \forall \varepsilon > \varepsilon _p, \sigma \propto \varepsilon \end{aligned}$$

With $\varepsilon _p$ being the characteristic strain value that delimits the elastic and the plastic regime of the stent. The coefficients that were used are extracted from Antonini et al. (2021) (Table 4).

**Table 4:** Stent material properties

Young’s modulus (GPa)	Poisson’s ratio (-)
(a) Elastic properties	
203	0.3

Plastic stress (MPa)	Plastic strain (-)
(b) Plastic properties	
480	0
1208	0.35
1300	0.62

The angioplasty balloon has a nominal diameter of $3 \, \text {mm}$, matching the healthy diameter of the coronary artery models. This balloon was assumed to exhibit a first-order Ogden hyperelastic behavior (Poletti et al. 2022). The dimensions of the stent and the balloon are based on clinically used angioplasty tools (Dong et al. 2018).

4 $$\begin{aligned} \Psi = \frac{2\mu }{\alpha }\Bigl (\lambda _1^\alpha +\lambda _2^\alpha +\lambda _3^\alpha -3\Bigr ) \end{aligned}$$

The coefficients used in Eq. 4 are detailed in Table 5. Before the stent was crimped, the balloon was folded into a tri-folded configuration.

**Table 5:** Balloon material properties

$\mu \ (-)$	$\alpha \ (-)$
80	− 15

**Table 6:** Artery material properties

	$C_{10}$ (MPa)	$C_{20}$ (MPa)	$C_{30}$ (MPa)	$C_{40}$ (MPa)	$C_{50}$ (MPa)	$C_{60}$ (MPa)
(a)Elastic properties						
Media	$7.21\cdot 10^{-2}$	$3.71\cdot 10^{0}$	$-1.56\cdot 10^{2}$	$9.18\cdot 10^{3}$	$-2.61\cdot 10^{5}$	$2.91\cdot 10^{6}$
Adventitia	$2.60\cdot 10^{-1}$	$4.76\cdot 10^{1}$	$-4.09\cdot 10^{3}$	$5.29\cdot 10^{5}$	$-2.69\cdot 10^{7}$	$5.65\cdot 10^{8}$

	Plastic strain (-)	Plastic stress (MPa)
(b)Plastic properties		
Media	0	0.7
	0.07	1.1
	0.4	2.0
Adventitia	0	1.6
	0.07	2.3
	0.4	4

The artery was 40*mm* long with an inner healthy diameter of 3*mm* and modeled as consisting of two layers: the outer adventitia and the media, measuring 0.34*mm* and 0.32*mm*, respectively, based on the measurements made on 13 fresh human cadaveric hearts by Holzapfel et al. (2005).The intima layer was not modeled as a single tunica due to its minimal mechanical influence (Poletti et al. 2022). Instead, it was modeled as fused with the tunica media, forming together a single layer (Fig. 3). The artery is stenosed (in area) by 70%, according to the most severe conditions exhibited in OCT pullback analysis from patients with CAC given the size of the crimped angioplasty system (Table 1b). Both artery layers were assumed to exhibit hyperelastic behavior, governed by a 6th-order reduced polynomial strain energy density function (Poletti et al. 2022). The coefficients for this model are provided in Table 6.

The stent was meshed using first-order brick elements; while, the balloon was meshed with 4-node quadrilateral membrane elements. The overall artery model was constructed using the methodology described in Kadry et al. (2021) to create a 3D multi-material representation of atherosclerotic plaques from idealized CAD artery geometries.

### Simulations

From each intact calcium model ($270^{\circ }$ and $90^{\circ }$), we designed 22 models that incorporate all the possible variations of the following:

- $l \in \left\{ 1/3; 2/3\right\}$ and $w \in \left\{ 1/3; 2/3\right\}$ for the sided IVL-ID model
- $l \in \left\{ 1/3; 2/3\right\}$ and $w \in \left\{ 1/3; 2/3\right\}$ for the centered IVL-ID model
- $l \in \left\{ 1/3; 2/3\right\}$ and $w = 1$ for the through IVL-ID model

All models underwent virtual stenting using the same angioplasty system under the same boundary conditions.

**Fig. 4:**
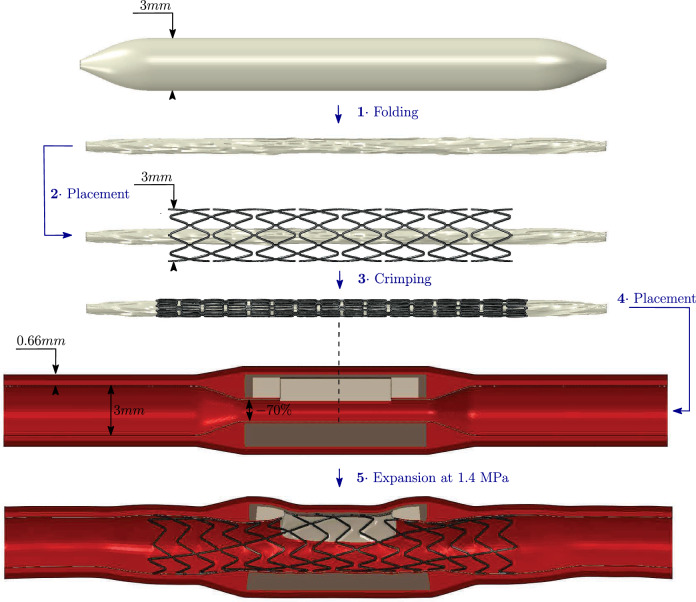
Overall pipeline showing the stent, balloon and artery used for our simulations. The same balloon and crimped stent were used for all the cases. The atherosclerotic portion of the artery is modeled with a stenosis of 70%. After being crimped, the stent is expanded in each virtual lesion with an inflation pressure of 1.4 MPa

Prior to expansion, the angioplasty balloon was folded into a tri-fold configuration using rigid cylinders. Subsequently, the stent was crimped onto this balloon. Rigid planes encircled the stent and were subjected to radial inward displacement to crimp the stent in place. Refer to Fig. 4 for the final setup after expansion.

Both the proximal and distal ends of the artery and balloon were pinned. The balloon was inflated to a pressure of 1.4 MPa ($\approx 14$ atm). The stent length was deliberately chosen to overlap both healthy ends of the artery, ensuring it spans the entire lesion. Given the highly nonlinear dynamics of this simulation, which involves contact and significant deformation, Abaqus/Explicit was utilized. Quasi-static conditions were ensured by limiting the total kinetic energy to below 5% of the total potential energy. The contact between all components was assumed to be hard and frictionless. Mass scaling algorithm (a target time increment of $5 \cdot 10^{-8} s$) was used.

For this study, we define the clinical outcome as the lumen gain $\alpha$, as described in Eq. 5:

5 $$\begin{aligned} \alpha (u) = A_a(u)-A_b(u) \end{aligned}$$

With: $u=z/L_a$, *z* is the axis of the artery. $A_a(u)$ is the surface area of the lumen at the position *u* after stenting, and $A_b(u)$ is the cross sectional area of the lumen at the position *u* before stenting. The variable $\alpha$ was measured for all simulations along the artery. To compare the results for the $90^{\circ }$ and $270^{\circ }$ arc angle lesions, we define the relative lumen gain $\Lambda$ for each IVL-ID model, where the lumen gain is $\alpha$ along the artery, as:

6 $$\begin{aligned} \Lambda = \mathop {\textrm{avg}}\limits _{u \in lesion} \alpha (u)-\bar{\alpha }(u) \end{aligned}$$

Here, $\bar{\alpha }$ represents the lumen gain of the corresponding artery model without defect (i.e., the corresponding intact model). $\Lambda$ can thus serve as a first-order metric indicating the extent to which the IVL-ID has benefited stent expansion, relative to an expansion of an intact artery. The $\Lambda$ values are presented in Table 7.

**Table 7:** $\Lambda$ values for each IVL-ID model

*l*	*w*	$\Lambda$ (%)
(a)Sided model, $\theta =90^\circ$		
1/3	1/3	1.0
1/3	2/3	3.0
2/3	1/3	9.7
2/3	2/3	16

*l*	*w*	$\Lambda$ (%)
(b)Centered model, $\theta =90^\circ$		
1/3	1/3	1.9
1/3	2/3	1.8
2/3	1/3	5.7
2/3	2/3	17

*l*	*w*	$\Lambda$ (%)
(c)Through model, $\theta =90^\circ$		
1	1/3	20
1	2/3	45

*l*	*w*	$\Lambda$ (%)
(d)Sided model, $\theta =270^\circ$		
1/3	1/3	4.0
1/3	2/3	6.5
2/3	1/3	16
2/3	2/3	25

*l*	*w*	$\Lambda$ (%)
(e)Centered model, $\theta =270^\circ$		
1/3	1/3	7.9
1/3	2/3	8.5
2/3	1/3	16
2/3	2/3	29

*l*	*w*	$\Lambda$ (%)
(f)Through model, $\theta =270^\circ$		
1	1/3	19
1	2/3	51

Finally, to get a sense of whether the length or the width is the main factor influencing post-stent lumen gain, for each artery sub-model X (with $X=S$ for the sided model, $X=C$ for the centered model and $X=T$ for the through model), for each one of the calcified plaque arc angles, we define a first-order influence coefficient for the length on lumen gain $\Delta _l$ as:

7 $$\begin{aligned} \Delta _l^X = \frac{\delta _l^X(2/3)+\delta _l^X(1/3)}{2} \end{aligned}$$

With:

$$\begin{aligned} \delta _l^X(x)=\frac{\Lambda (w=x,l=2/3)-\Lambda (w=x,l=1/3)}{1/3} \end{aligned}$$

For the through model, the values taken by $\Lambda$ change accordingly. We define the analog parameters for the width *w*. $\Delta _l^X$ and $\Delta _w^X$ quantify the influence of the width *w* and the length *l* on the lumen gain for the sub-model X. Table 8 shows how IVL increased post-stent lumen gain with respect to an expansion without IVL-ID.

Then, inspired by the concept of gradient, we compute $\Gamma _X$ as:

8 $$\begin{aligned} \Gamma _X = \left\| \begin{pmatrix} \Delta _l^X \\ \Delta _w^X \end{pmatrix} \right\| = \sqrt{\left( \Delta _l^X\right) ^2 + \left( \Delta _l^X\right) ^2} \end{aligned}$$

$\Gamma _X$ can be considered a measure of how IVL is beneficial to the sub-model X. The bigger $\Gamma _X$, the more beneficial IVL is for the sub-model X.

Lastly, we define $\mu _{90}$ as follows:

9 $$\begin{aligned} \mu _{90}= \frac{\Gamma _S+\Gamma _C+\Gamma _T}{3} \end{aligned}$$

And similarly, $\mu _{270}$ is defined. $\mu _{90}$ and $\mu _{270}$ are global metrics of how much IVL was beneficial in improving lumen gain for the $90^{\circ }$ and the $270^{\circ }$ plaques, taking into account the 3 sub-models for each plaque extension. The models have in total $\approx$ 1 million elements. The simulations were performed with $\approx$ 100 Intel(R) Xeon(R) CPU E5-2650 v3 @ 2.30GHz CPUs and lasted $\approx$ 20 h.

## Results and discussion

This study assessed the efficacy of IVL in preparing heavily calcified coronary lesions for stent deployment. Specifically, it sought to investigate how variations in defect morphology and position induced by IVL affect stent expansion. The effectiveness of IVL was evaluated by comparing the lumen area, stress distribution across calcified plaque and strain distribution across the lesion post-stent deployment across different lesion morphologies and defect severities. Results are presented for the three lesion models: sided, centered, and through, with each model tested under two conditions of calcium arc angles, $\theta _c=90^\circ$ and $\theta _c=270^\circ$.

**Fig. 5:**
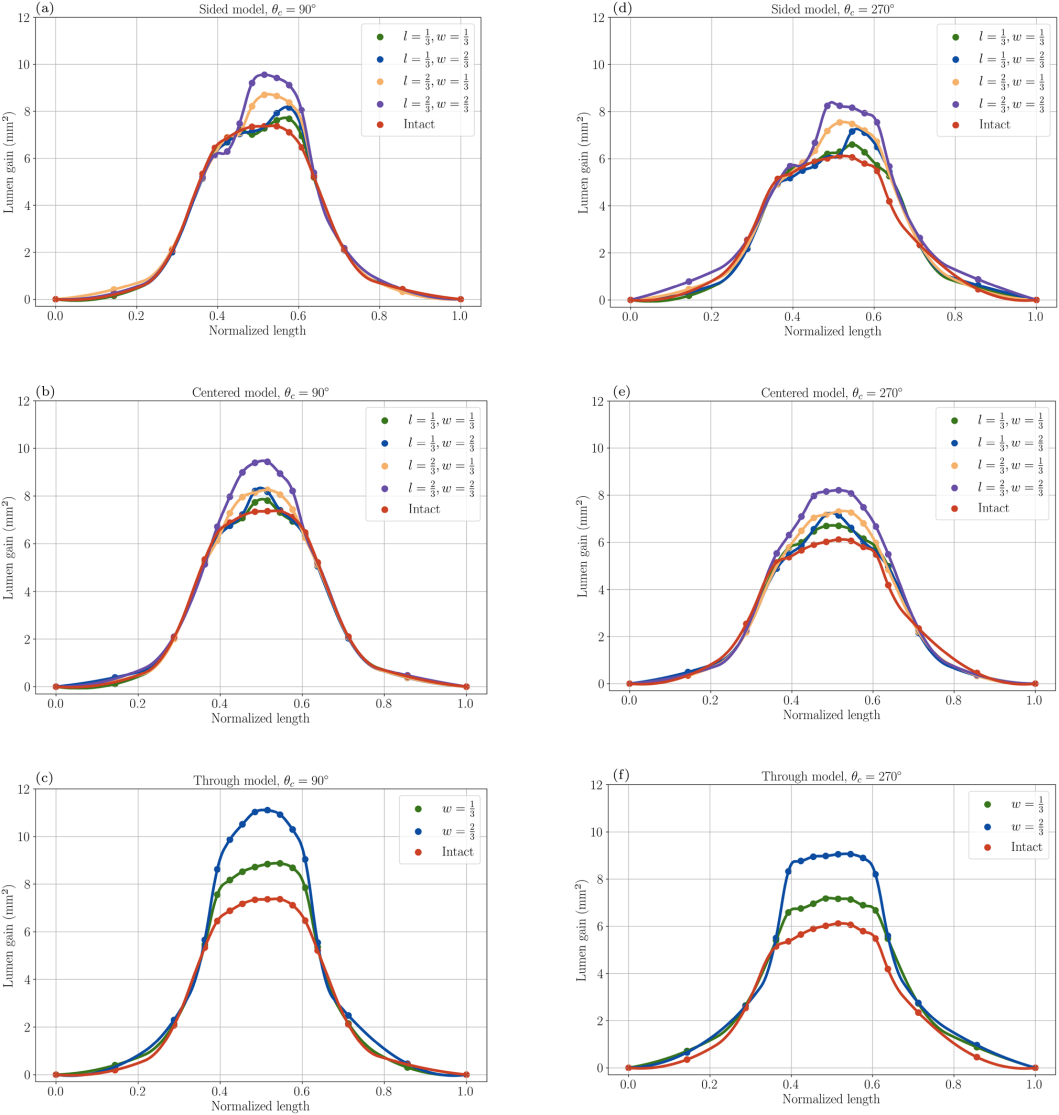
Lumen gain along the length of the artery for all models. Figures 5a through 5c and Fig. 5d through 5f are, respectively, lumen gains for the $90^{\circ }$ and $270^{\circ }$ arc angle lesions

All the results (Fig. 5) exhibit a similar trend. The impact of the defect is primarily local, underscoring the importance of targeted IVL application.

Our analysis indicates that the location of the defect along the arterial segment does not significantly impact the stent expansion, 5a, b, c and d), along with their corresponding $\Lambda$ values (Table 7a, b, e and d). This suggests that the primary determinant of lumen gain is the size of the defect rather than its position, regardless of plaque size. This induces that making sure of a precise calibration of the location of the IVL lithotripters with respect to the calcified lesion does not seem to be needed. Also, with the values of Table 8a, we can get the average of the $\Gamma _X$ values for the sided and centered sub-models:

10 $$\begin{aligned} <\Gamma _S>= &  \frac{\sqrt{33^2 + 12^2}+\sqrt{46^2 + 17^2}}{2}\approx 83 \end{aligned}$$

11 $$\begin{aligned} <\Gamma _C>= &  \frac{\sqrt{29^2 + 17^2}+\sqrt{43^2 + 20^2}}{2}\approx 81 \end{aligned}$$

The relative error being 2%, this corroborates the idea that the location of the IVL-ID does not play an important role for stenting outcomes improvement.

Lesions with end-to-end defects (Fig. 5c and e and Table 7c, f) appear to yield the best stenting outcomes overall across both small ($90^{\circ }$) and large ($270^{\circ }$) calcified plaques (Table 8b). This highlights the importance of using IVL to fragment heavily calcified plaques into smaller pieces and leveraging the plaque discontinuity to maximize post-stent lumen area. The most effective strategy to optimize lumen gain with IVL is therefore to induce large, fully penetrating IVL-ID (through models). Centered defects are also effective, particularly in wider plaques, likewise for sided IVL-IDs. The differences between the $\Lambda$ values of Table 7e, d; and Table 7b, a are minimal.

**Table 8:** $\Delta _l^X$ and $\Delta _w^X$ values for all the IVL-ID sub-models with $X=T$ (through sub-model), $X=C$ (centered sub-model) and $X=S$ (sided sub-model)

	Sided		Centered		Average	
	$\Delta _l^S$	$\Delta _w^S$	$\Delta _l^C$	$\Delta _w^C$	$\Delta _l^X$	$\Delta _w^X$
(a)$\Delta _l^X$ and $\Delta _w^X$ values for IVL-ID sided and centered sub-models with $X=C$ and $X=S$. In the split cells, the right-hand value is $\Delta _w^X$ and the left-hand value is $\Delta _l^X$						
$\theta _c = 90^{\circ }$	33	12	29	17	31	14.5
$\theta _c = 270^{\circ }$	46	17	43	20	44.5	18.5
Average	39.5	14.5	36	18.5		

	Through
(b)$\Delta _w^T$ values for the IVL-ID through sub-model	
$\theta _c = 90^{\circ }$	75
$\theta _c = 270^{\circ }$	96
Average	85.5

On the other hand, the size of the defect appears to be crucial for non-transpiercing IVL-IDs. The $\Lambda$ values (Table 7a, b, e and d), consistently follow the same trend regardless of the defect’s location: the smallest defect corresponds to the smallest $\Lambda$, while the biggest defect maps to the highest $\Lambda$. For cases in between, inducing a long and narrow IVL defect appears to be more beneficial than creating a short and wide one - irrespective of the IVL-ID location and calcified plaque size.

**Fig. 6:**
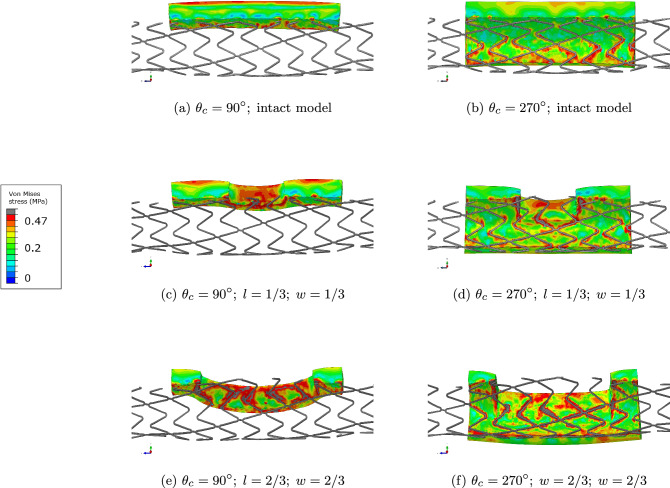
Stress map in MPa of the intact (Fig. 6a, b), $90^{\circ }$ (Fig. 6c, e) and $270^{\circ }$(Fig. 6d, f) calcified plaques of the centered IVL-ID models. Calcified plaque yield stress is 0.48 MPa. We can observe the stent imprint on the calcified plaque, which represents yield

**Fig. 7:**
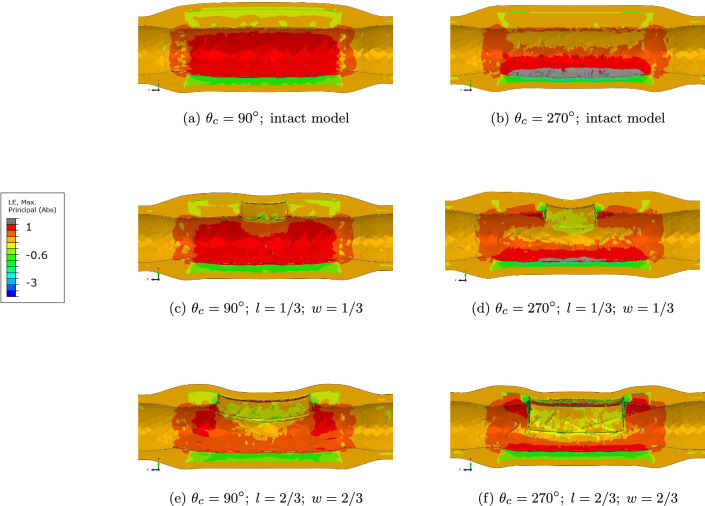
Strain map of the lesions containing the intact (Fig. 7a, b), $90^{\circ }$ (Fig. 7c, e) and $270^{\circ }$ (Fig. 7d, f) calcified plaques of the centered IVL-ID models

This tendency is confirmed by Table 8: the length *l* of the IVL-ID seems to be the most influential parameter for angiographic outcomes. Indeed, using the last row of Table 8a:

12 $$\begin{aligned} \frac{39.5+36}{2} > \frac{14.5+18.5}{2} \end{aligned}$$

This suggests that even though IVL-ID that split calcified plaques map to the best stenting outcomes, among non-transpiercing IVL-IDs, the longer ones will be more beneficial for lumen gain. This insight could guide future IVL applications to prioritize creating elongated defects over broad ones to optimize stent deployment and subsequent clinical outcomes. Our study shows that IVL is advantageous for both small and large plaques, but more for large plaques. Indeed, Table 9 shows that $\mu _{270} > \mu _{90}$. This corroborates the fact that IVL is usually performed in heavily calcified lesions.

**Table 9:** $$\mu _{90}$$ and $$\mu _{270}$$

$\mu _{90}$	$\mu _{270}$
144	192

The stress maps (Fig. 6) highlight the impact of calcified plaque geometry and IVL-ID dimensions on stress distribution during stent expansion. The stress values and patterns in the plaque resulting from our simulation are consistent with the ones in the literature (Conway et al. 2014). The regions of highest stress are located at the boundary of the calcified plaques, more specifically at the stent/lesion interface. The interior of the calcified plaque remains in the elastic regime. For small plaques (Fig. 6c, e), the stress concentrates mainly in and in the vicinity of the IVL-ID. For large plaques (Fig. 6d, f), the observations are similar: Stress is highly localized around the stent strut contact points, with peak values observed near the edges of the IVL-IDs (Fig. 6c, e, d and f). Persistent high-stress regions suggest a potential risk for localized plaque damage, emphasizing the importance of considering plaque geometry and defect propagation during IVL.

For both small $90^{\circ }$ plaques (Fig. 6a, c and e) and large $270^{\circ }$ plaques (Fig. 6b, d and f), as the IVL-ID gets bigger, stress redistributes but remains concentrated around the struts located in the vicinity of the IVL-ID. Since the IVL-ID creates non-contiguity within the calcified plaque, it also creates stress concentration prone regions that are not present in the intact model, i.e., the boundary of the IVL-ID. The following stenting procedure makes the stress values in these regions go up to the yield value of 0.48 MPa for calcium (Fig. 6).

It also seems that the bigger the IVL-ID, the more calcified plaque is close to yield—which makes sense since the IVL-ID creates additional surfaces within the plaque for the stent to expand against, thus damaging more the plaque. This can be linked to the fact that large IVL-IDs map to higher lumen gains. Indeed, in our study, calcium is modeled as perfectly plastic, meaning as it enters its plastic state, there is theoretically no effort required to further deform it (usually, materials are less rigid when they exhibit plastic behavior, and for this study we chose the ideal case when the yielded material is not rigid at all). Yielded regions are thus easier to expand than elastic regions. Additionally, the bigger the IVL-ID, the less hard material there is for the stent to push against to expand the artery, i.e., the easier it is for the stent to expand. Both effects combined together indeed explain the results shown in Table 8 or the plots in Fig. 5 for large IVL-IDs.

For both plaque size, given the localized stiffening due to calcium, the remaining portion of the vessel underwent higher stretch (Fig. 7), which could be associated with potential future complications such as re-occlusion due to neointimal hyperplasia. In case of an IVL producing a bigger defect in the same lesion, strains decrease in the arterial wall, especially at the site diametrically opposed to the lesion and at the location of the IVL-ID (Fig. 7c compared to Fig. 7e; and 7d compared to 7f). It corroborates IVL sustained safety and efficacy that has been demonstrated by long-term follow-up data with low rates of MACE, target lesion revascularization, and stent thrombosis (Kereiakes et al. 2022; Saito et al. 2022). Similar behavior is observed when considering the creation of an IVL-ID (Fig. 7a compared with Fig. 7c, b compared with Fig. 7d). This shows a substantial benefit of IVL as this decrease in strain can be related to lower risk of restenosis (Zhao and Gu 2012).

It is worth noting that the stress map was added only for a comparison purpose: The stress values given by one simulation cannot be interpreted when considered as absolute; they are only meaningful when compared to the stress values from other simulations, even though our stress results are comparable with the literature (Conway et al. 2014).

The results mentioned above, although promising, need to be interpreted within the context of certain limitations inherent in our study methodology. Specifically, the calcified plaque in our models was treated as an elastic–plastic material without incorporating a fracture model. This simplification likely impacts the accuracy of the results, as real calcified plaques may exhibit fracture and other complex behaviors under stress during stent expansion that are not captured by purely elastic–plastic assumptions. Incorporating a damage model in future simulations could offer a more nuanced understanding of the biomechanical interactions between the stent and the lesion, potentially leading to more precise predictions of clinical outcomes.

Moreover, the current study employed idealized straight artery geometries rather than patient-specific models (Straughan et al. 2023; Kadry et al. 2023, 2024, 2022). While this approach facilitates controlled comparisons and aids in understanding general trends, it may not fully capture the anatomical complexities observed in individual patients. Transitioning to patient-specific artery geometries in future research will enhance the applicability and relevance of our findings. By incorporating detailed anatomical features such as vessel curvature or multi-material interactions, such analysis would enable the development of more tailored and effective IVL strategies. This will potentially improve personalized treatment plans for patients with heavily calcified coronary artery disease.

In this study, the plaque components were assumed to have no microscopic IVL-induced defects. However, IVL is known to induce micro-defects within the plaque, potentially altering the mechanical behavior of the lesion components. These micro-defects can influence the structural integrity and stress distribution within the plaque, which may affect the outcomes of IVL treatment. Future work should consider incorporating the impact of these induced micro-defects by modifying the material properties of the plaque components to more accurately reflect the post-IVL state and improve the predictive accuracy of the model. Finally the assumption of concentric lesions was made. In reality, many lesions tend to be eccentric rather than perfectly concentric, which can greatly influence both the biomechanical behavior of the arterial wall in response to stenting. The stress distribution and plaque fracture dynamics could vary substantially in eccentric lesions, potentially affecting clinical outcomes.

## Conclusion

We have demonstrated the substantial impact of defect morphology on stent expansion post-IVL, wherein larger defects promote greater lumen gain, irrespective of whether the calcified plaques are small ($90^{\circ }$ arc angle) or more extensive ($270^{\circ }$ arc angle). However, IVL appears to offer more significant benefits for larger calcified lesions. Notably, the influence of these defects does not scale linearly with their length and width. Additionally, the study suggests that the location of the IVL-induced defect (whether it is centered or sided) may not significantly affect lumain gain. Lastly, the length of the IVL-ID seems to have a bigger influence on the post-stent lumen gain than the width. These findings underscore the potential of IVL to enhance lesion compliance and improve clinical outcomes in PCI. Moving forward, future studies must incorporate damage mechanics and patient-specific geometries to enable more personalized treatments for patients with severe calcified coronary arteries.

## Data Availability

No datasets were generated or analyzed during the current study.
